# Alignment-free supervised classification of metagenomes by recursive SVM

**DOI:** 10.1186/1471-2164-14-641

**Published:** 2013-09-22

**Authors:** Hongfei Cui, Xuegong Zhang

**Affiliations:** 1 Department of Automation, Bioinformatics Division/Center for Synthetic & Systems Biology, TNLIST, MOE Key Laboratory of Bioinformatics, Tsinghua University, Beijing 100084, China; 2 School of Life Sciences and School of Medicine, Tsinghua University, Beijing 100084, China

**Keywords:** Metagenome, Classification, K-tuple, R-SVM, Alignment-free, Sequence signatures

## Abstract

**Background:**

Comparison and classification of metagenome samples is one of the major tasks in the study of microbial communities of natural environments or niches on human bodies. Bioinformatics methods play important roles on this task, including 16S rRNA gene analysis and some alignment-based or alignment-free methods on metagenomic data. Alignment-free methods have the advantage of not depending on known genome annotations and therefore have high potential in studying complicated microbiomes. However, the existing alignment-free methods are all based on unsupervised learning strategy (e.g., PCA or hierarchical clustering). These types of methods are powerful in revealing major similarities and grouping relations between microbiome samples, but cannot be applied for discriminating predefined classes of interest which might not be the dominating assortment in the data. Supervised classification is needed in the latter scenario, with the goal of classifying samples into predefined classes and finding the features that can discriminate the classes. The effectiveness of supervised classification with alignment-based features on metagenomic data have been shown in some recent studies. The application of alignment-free supervised classification methods on metagenome data has not been well explored yet.

**Results:**

We developed a method for this task using k-tuple frequencies as features counted directly from metagenome short reads and the R-SVM (Recursive SVM) for feature selection and classification. We tested our method on a simulation dataset, a real dataset composed of several known genomes, and a real metagenome NGS short reads dataset. Experiments on simulated data showed that the method can classify the classes almost perfectly and can recover major sequence signatures that distinguish the two classes. On the real human gut metagenome data, the method can discriminate samples of inflammatory bowel disease (IBD) patients from control samples with high accuracy, which cannot be separated when comparing the samples with unsupervised clustering approaches.

**Conclusions:**

The proposed alignment-free supervised classification method can perform well in discriminating of metagenomic samples of predefined classes and in selecting characteristic sequence features for the discrimination. This study shows as an example on the feasibility of using metagenome sequence features of microbiomes on human bodies to study specific human health conditions using supervised machine learning methods.

## Background

Microbes play important roles in human health and habitat environment. There are a large amount of microbes accreting with human, forming many microbiomes on human bodies such as on human skins [1-3], mouths [4,5] and bowels [6-9]. The total number of human genes is only about 1% of the number of genes of all microbes on a human body [10]. The study of microbiomes is very important because they may have large impact on human health such as on the immune system, metabolism and nutrition, and some microbes may be important pathogens with great virulence [11]. For example, people are interested in which microbes have pathogenicity and what kinds of microbiotas are harmful or beneficial to human. But most microbes are difficult to be cultured. The study of metagenomics which sequences genomes directly from the mixture of multiple microbes allows us to study mixture of microbes without culturing each of them separately. Using metagenomic data to compare and classify different kinds of microbiome samples is an important new approach for studying microbiomes.

The 16S rRNA sequencing approach is a conventional way of identifying microbes and many researchers have used this method in comparing microbome samples [1-10,12]. The 16S rRNA genes or their hyper-variable regions of organisms are sequenced and mapped to known 16S rRNA databases (e.g. RDP [13], Greengene [14], SILVA [15,16], EzTaxon-e [17]). Then, the catalogue of microbes for each sample can be built. Comparing microbe catalogues between different samples can give us information about different compositions of microbe communities. A major limitation of this approach is that it can only analyze microbes with known 16S rRNA sequences. Another way to get the taxonomic catalogue is to cluster 16S rRNA fragments into OTUs (operational taxonomic units) to compare 16S rRNA datasets without using references (e.g., [18-22]). But the information that 16S rRNA can represent is still limited, especially with regard to the functional elements that are characteristic to the microbiomes. Metagenome sequences contain much more information than 16S rRNA sequences, especially about the genes of all microbes in the community, which can help us to understand the microbes’ potential functions and interaction with environments or their hosts. With the development of next-generation sequencing (NGS) techniques, more and more metagenomic data have been generated (e.g., [23-27]). This brings more opportunity for investigating the relationship between microbiomes and their habitats.

The basic approach for analyzing metagenome data is alignment-based methods. NGS reads are first mapped to known datasets of microbial genomes and genes (e.g., the NCBI nr database and/or the KEGG database), and a catalogue of taxonomy or genes for each metagenomic sample is obtained. Comparison of the metagenome samples can be done based on the catalogues or abundances of known microbial genes. Both supervised and unsupervised machine learning methods can be applied on such data to classify samples into informative classes or to find intrinsic clusters in the samples. The limitation of these types of methods is also obvious. Firstly, reference databases of microbial genomes and genes are still far from complete. Known microbial genome sequences only occupy a small part of the whole microbial world. Secondly, the alignment of the huge amount of short reads to multiple references often means a high complexity of computing. Alignment-free methods are therefore a promising alternative approach for analyzing massive metagenome data.

Alignment-free methods based on sequence signatures are powerful for analyzing genomic data without using reference genomes. The basic strategy is to profile the composition of short oligonucleotides (k-mers, k-tuples) as signatures for distinguishing sequences from different organisms [28-30]. Such sequence signature features can be counted directly from the NGS short reads without the need of assembly or mapping. Similarity measures can be defined on sequence signatures and can be used to study the beta-diversity of metagenome samples. For example, Willner et al [31] analyzed di-, tri- and tetra-nucleotide abundances of 86 microbial and viral metagenomes, then used PCA (principal component analysis) and hierarchical clustering to show “definitive groupings of metagenomes drawn from similar environment”. Ghosh et al [32] developed a method called HabiSign which uses tetra-nucleotide patterns of metagenomic sequences to cluster the samples at biome, phenotypic and species levels. Jiang et al [33] studied several major dissimilarity measures with sequence signatures and their performances on the comparison of metagenome samples. These studies all belongs to the unsupervised learning strategy, which aims to reveal similarity/dissimilarity and grouping relationships among the studied samples. This strategy is powerful for discovering major intrinsic clustering relations among the compared samples and have been widely adopted in many studies. However, in some other scenarios, one may be interested in studying two predefined classes or comparing samples of two given groups. For example, we may want to compare human microbiome samples of people suffering a certain type of disease and that of healthy controls with metagenome sequence data. This is similar to cancer classification studies with gene expression data. Typically the classes of interest may not be the dominating assortment in the data, and therefore may not be revealed as separated clusters in the unsupervised comparison. Supervised pattern recognition methods for classification and feature selection are needed for such tasks. With sequence signatures as candidate features, we want to build a learning machine or classifier to classify samples into the predefined classes using samples with known classification labels as training data. Another equally important task is to select sequence features that enable the classification. If the classification performance is reasonable, the selected sequence features could be potentially used as microbial markers for the disease. Such markers also provide hints for investigating the association and involvement of microbes or genes in the human disease.

Supervised classification has been successfully applied on 16S rRNA data and on alignment-based features of metagenomic data in recent years [34-38]. However, the alignment-free supervised classification strategy with sequence signatures on metagenome data with regard to predefined classification goals has not been well documented yet. In this study, we investigated the feasibility of using k-tuple sequence signatures for supervised classification of metagenome samples. Table 1 shows the major categories of machine-learning applications on the analysis of microbiome data. This study fills in the category of alignment-free supervised classification of metagenome data. We adopted the Recursive SVM (R-SVM) method we developed for gene and protein expression data [39], which can perform feature selection and classification in a wrapped manner. When the sample size is small but the number of candidate features is large, supervised learning methods can face the risk of overfitting the training data. The R-SVM method has been designed for avoiding possible overfitting in the learning, and we applied stringent leave-one-out cross-validation (LOOCV) and permutation experiment to evaluate the classification accuracy and statistical significance. We first experimented with a set of simulation data. Then a dataset of 10 real tree genomes from two families [40] was tested as a real but simplified case of metagenomic data. Finally we applied the method on a real metagenome dataset [25] of 124 European individuals to study the classification of inflammatory bowel disease (IBD) patients from normal controls based on metagenome features of their fecal microbiomes. Satisfactory leave-one-our cross-validation accuracy and test accuracy was achieved. The work illustrates the feasibility of using supervised classification methods on sequence signatures to study specific classifications of metagenome samples. It opens a promising new approach for analyzing massive NGS short reads data of metagenome samples for properties that may not be revealed by unsupervised cluster analysis.

**Table 1:** Major categories of machine-learning methods for analysis of microbiome samples with sequencing data

**Data type**	**Metegnome sequencing**				**16S rRNA**
Feature type	**Alignment-free**		Alignment-based		OTU-based or microbial taxon-based
	(based on sequence signature features without using any database)		(based on features obtained by mapping sequence reads to annotation databases)		
Type of machine learning	**Supervised classification**	Unsupervised clustering	Supervised classification	Unsupervised clustering	Supervised or unsupervised

The highlighted category (alignment-free supervised classification of metagenome data) is that of the current work.

## Methods

### Feature extraction and classification

The basic idea of alignment-free methods is to use the occurrence or frequency of k-tuples (k-mers, k-grams) as sequence signatures of the studied genomes or metagenomes, and use such signatures as features for the clustering or classification. Existing works using unsupervised clustering approaches have shown that such sequence signatures contain information that can separate metagenomes of different characteristics into meaningful groups, and can cluster genomes according to their phylogenetic relations [30,41,42]. For supervised classification of metagenomes, we want to study whether and how k-tuples features can be used to separate metagenome samples into predefined classes that we are interested in. This is important in exploring whether a certain phenotypic character of the host (e.g., a particular human disease) is associated with metagenome features of the microbiome on the human body.

### Counting k-tuple features

The k-tuple frequency is the relative occurrence frequency of each word of k nucleotides in a metagenome sample. This can be counted directly from the NGS short reads of the metagenome data, without the need of full or partial sequence assembly. For a fixed length k of k-tuples, all 4^*k*^words are taken as the candidate features and their occurrences are scanned and counted in every short read. Summing up all counts gives the total count vector of dimension4^*k*^, with each component as the count of the corresponding k-tuple word. We standardized the vectors in two steps: The frequencies of all k-tuples in each sample were normalized by the total count of k-tuples to remove the effect of different sequencing depths between samples; then for each feature, the component values across all samples were standardized to mean = 0, variance = 1.

### Feature selection and classification

Recursive SVM (R-SVM) [39] is a modified support vector machine algorithm which performs feature selection while building the classifier in a multiple-step recursive manner following a given descendant ladder. To lower the risk of overfitting, the basic linear kernel is used in the SVM to keep the least model complexity for situations when sample size is small but feature dimension is high. At each level of feature selection, R-SVM first applies the SVM on all available features. The decision function is of the form:

$$\begin{array}{l} g\left(x\right)=\mathrm{sgn}f\left(x\right)=\mathrm{sgn}\left\{\left(w\cdot x\right)+b\right\}\\ \;=\mathrm{sgn}\left\{\sum_{i=1}^{n}\alpha_{i}y_{i}\left(x_{i}\cdot x\right)+b\right\} \end{array}$$

where *n* is the number of samples in the training set, **x** is the feature vector of a test sample, **x**_*i*_ is the vector of training sample i and *y*_*i*_ ϵ { − 1, 1} is the corresponding class label. The parameters *α*_*i*_’s and *b* are trained from the training dataset by maximizing the separation margin and minimizing the prediction error on training data. And the weight vector of the features is $w=\sum_{i=1}^{n}\alpha_{i}y_{i}x_{i}$. The weight of each feature in the vector can be regarded as the contribution of the feature in the trained classifier. Then features are ranked according to their differences between the two classes weighted by their weights in the trained SVM, and a number of features from the top of the rank will be selected for the classification and feature selection at the next level. The details of the method was described in [39] in the context of gene/protein expression study. In this work, the features are the standardized frequencies of the k-tuple words, and the feature selection ladder was set as {All, 1000, 500, 200, 100, 50, 30, 20, 10, 5}, which means that we start from all available features, and select the top 1000, 500, …, 5 features at each recursive step. When *k* is small, the number of all features can be less than 1000. We then start with all features and use the biggest number in the list that is smaller than the number of features in the next selection.

To assess the classification performance at each feature selection level and to choose the level of best performances, we use the accuracy of leave-one-out cross validation (LOOCV) to evaluate the result of classification and feature selection. In each round of LOOCV, one sample is left out and R-SVM is trained on the remaining samples. At each feature selection level, an SVM model is trained and is used to predict the class label of the left-out sample. After all the samples are left-out once, the rate of correct prediction is calculated at each level of feature selection. It should be noticed that in this procedure, feature selection must also be included in the validation step. That is, the test sample should be left out before any feature selection, otherwise the assessment of the performance can be biased and over-optimistic [39], which can also be regarded as overfitting caused by improper timing of feature selection. We adopted the stringent way of LOOCV (called CV2 in [39]) in our experiment to give an unbiased assessment of the feature selection and classification performance. This procedure results in multiple sets of selected features at each level, so after we complete the cross-validation procedure, we run R-SVM on all samples again to obtain a unique set of feature selection results and a unique classification model that can be used to predict new samples. (There can be other voting-based ways of building the final R-SVM classifier after the cross-validation as described in [39], but we chose this simpler way in the current work as the the purpose is to study the feasibility of the approach.)

### Permutation test

For a particular classification question defined on a phenotype property of the host of the microbiome, such as a specific human disease, it is not sure whether there is an association between the classification and metagenome features. As the number of samples is small but the number of features is huge, chance alone may result in some apparent accuracy in the classification on some datasets. Therefore, it is necessary to check the statistical significance of the classification performance by comparing the accuracy obtained on the real data with the null distribution of accuracies on similar data when there is no association between the features and classification labels. We implemented a set of permutation experiments to answer the question in this study. In each experiment, 1000 times of permutations were applied on class labels of the samples, with labels randomly assigned to the samples while keeping the total number of each label consistent with the true labels. This is a way to generate multiple datasets with real data that have no association with the classification labels. The same R-SVM LOOCV procedure was applied on each permuted dataset, and the best LOOCV result on each permuted data was recorded. With 1000 permutations, we obtain an estimated null distribution of the classification performance when there is no true classification signal in the data. Comparing the performance we obtained on the true data to this null distribution, we obtain an estimation of the statistical significance of the achieved performance with the permutation p-value.

### Simulation data

**Figure 1:**
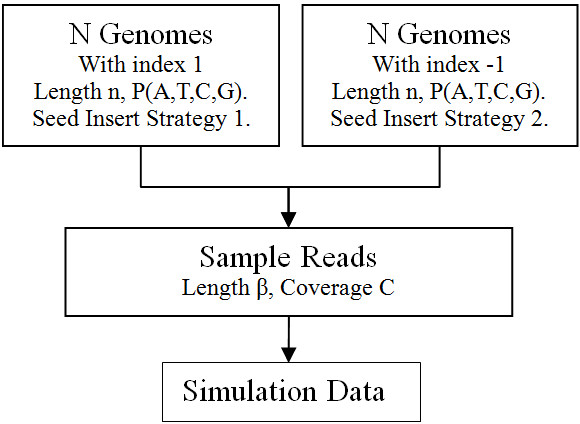
The flow chart of generating the simulation data.

We first created a series of simulated genomic data to study the performance of the method. The simulation data are summarized in Table 2. The simulation is to mimic the situation of two groups of genomes that are similar to each other but each group has its own characteristics in some sequence patterns. We used two steps to generate the data (Figure 1). Firstly, we randomly generate the genome sequences for two classes by inserting predefined k-tuple sequence patterns (seeds) in the random genomes, and then we sample short reads from the simulated genomes using the NGS simulation tool Metasim [43].

**Table 2:** Simulation experiments

**K-tuple**	**0.01**		**0.005**		**0.003**	
**Density**	**#kinds of seed**		**#kinds of seed**		**#kinds of seed**	
	**Group 1**	**Group 2**	**Group 1**	**Group 2**	**Group 1**	**Group 2**
Simulation1	0	3	0	3	0	3
	0	5	0	5	0	5
	0	10	0	10	0	10
Simulation2	1	1	-	-	-	-
	1	5	-	-	-	-
	5	5	-	-	-	-
	5	10	-	-	-	-
	10	10	-	-	-	-

The symbol “-” means that there is no experiment under the parameters.

In this step, there are two key elements: the background model and seed insertion strategy. We generated 2*N* genomes with *N* of them belonging to class 1 and the other *N* belonging to class 2. In the two classes, genome lengths and background models are the same, but sequence seeds are inserted with different strategies (seed sequences, numbers of seeds, lengths of seeds and densities of seeds).

We fix *N* = 25 with a uniform model in which *P*_*A*_ = *P*_*C*_ = *P*_*G*_ = *P*_*T*_ and genome length *n* = 10,000 for all simulated genomes. When inserting seeds of fixed length *l*, we randomly choose an insertion position *p*_*ins*_(1 ≤ *p*_*ins*_ ≤ *n* − *l* + 1), and replace the *l* letters from this position with the seed we want to insert. Each seed are inserted randomly for several times according to the seed density parameter. The density of one seed is defined as $\frac{l\times \#\mathrm{seed}}{n}$ where *l* is the length of the seed, #seed is how many times this seed is inserted into the genome and *n* is the length of the genome. Table 2 shows the 14 experiment groups of simulation data we generated. In each group, seed number and seed density are shown in the table and seed length *l* = 4, 5, 6, 7 are all experimented (a total of 14 × 4 experiments). All seed sequences are randomly generated, and the seeds we used for inserting are the same in each row of Table 2. Two types of simulations were designed. In Simulation 1 (Sim-1), one class is purely background sequences with no seed inserted, while another class has inserted seeds with varying seed density and the number of seed types. In Simulation 2 (Sim-2), both classes have seeds inserted, but with different seeds and parameters.

In the second step, we use Metasim [43] to randomly sample reads from a given sequences, mimicking the sequencing procedure of NGS. We used the following parameters: read length *β* = 50, coverage *C* = 10, and no sequencing error.

As the differences between the two classes of simulated data are known, we use these data to investigate how well the proposed method can discriminate the two classes with sequence features, and how well it can reveal the true differences (i.e., the seeds) between the two classes.

### Real genome data for tree family classification

To test how the proposed method performances on real data, we first applied it on a real tree genome dataset that has been used in [41], as a simplified example of metagenome data. It contains 10 trees of two families: 6 trees of the Fagaceae family and 4 trees of the Moraceae family. Fagaceae and Moraceae are two important tree families in the tropical rain forests of Southeast Asia [40]. The data are NGS short reads of 51 bp long sequenced by the Illumina platform and there is no complete genome sequence. The sequence sample information is given in Table 3. We want to study whether the unassembled genome sequence samples can be correctly classified to the two families with a small number of sequence signatures. All data are downloaded from NCBI (Data for Fagaceae: http://www.ncbi.nlm.nih.gov/sra?term=SRP001298; Data for Moraceae: http://www.ncbi.nlm.nih.gov/sra?term=SRP001299).

**Table 3:** Summary of the NGS short reads data of the tree genome data

**Group**	**SRX number**	**SRR number (run)**	**# of bases**
Fagaceae	SRX017683	SRR037802	1.2G
	SRX017436	SRR037484	0.816G
	SRX017340	SRR037437	1.4G
	SRX017339	SRR037158	0.979G
	SRX017338	SRR037157	1.2G
	SRX016680	SRR035946	1.1G
Moraceae	SRX017643	SRR037748	1.2G
	SRX017840	SRR038268	1.1G
	SRX017740	SRR037888	0.487G
	SRX017645	SRR037751	0.899G

### Real metagenome data for IBD classification

We applied our method on the metagenome data of faecal samples of 124 European individuals from [25] to classify inflammatory bowel disease (IBD) samples with control samples. The dataset contains metagenomic reads sequenced by Illumina GAIIx from faecal samples of 25 inflammatory bowel disease (IBD) patients and 99 individuals without IBD. We took them as the disease samples and the control samples. A total of 576.7 Gb of sequences were generated. Two settings were used in the original sequencing and therefore 14 samples have reads of 44 bp long, 109 samples have reads of 75 bp long, and one sample has both 44 bp reads and 75 bp reads. The average number of reads per sample is about 62 M. Reads with letter “N” were all chucked away in our experiments. The numbers of samples of the two classes are a little unbalanced, so we used the 25 IBD samples as the disease samples and randomly chose 25 control samples from the other samples in our LOOCV experiment. Then we also used these samples to train a classifier to be applied on the remaining 74 non-IBD samples as an independent test.

## Results and discussion

### Simulation results

#### The LOOCV accuracy

We did a total of 14 × 4 simulation experiments as described in Method and Data. In each experiment we used k-tuple features with length *k* varying between 3 and 8. Table 4 gives an example of one experiment. It shows the result on the data that no seed is inserted in group 1 and five seeds of length 6 are inserted in group 2 with the seed density 0.005. We can see that 100% accuracy can be obtained when we select 50, 30, 20, 10, and 5 features with k-tuple length of 6, when we select 1000, 500, 200, 100, 50, 30, 20 features with k-tuple length of 7, and when we select 20 features with k-tuple length of 5.

**Table 4:** The LOOCV error rates on the simulation data

**# of selected features**	**All**	**1000**	**500**	**200**	**100**	**50**	**30**	**20**	**10**	**5**
3-tuple	0.16	-	-	-	-	0.18	0.28	0.28	0.28	NaN
4-tuple	0.14	-	-	0.14	0.18	0.18	0.24	0.34	0.40	NaN
5-tuple	0.06	0.06	0.06	0.08	0.02	0.02	0.02	0.00	0.02	0.12
6-tuple	0.04	0.04	0.04	0.04	0.02	0.00	0.00	0.00	0.00	0.00
7-tuple	0.10	0.00	0.00	0.00	0.00	0.00	0.00	0.00	0.04	0.04
8-tuple	0.26	0.10	0.10	0.14	0.28	0.30	0.40	0.46	0.42	0.46

This table shows the LOOCV error rates when no seed is inserted in group 1 and five seeds of length 6 are inserted in group 2 with the seed density 0.005.

“# of selected” indicates the number of feature selected in each level. “features” indicates the k-tuple feature with the particular value of k. “all” is the situation when all the 4^k^ features are used. “-” is the feature selection level does not exist for the particular k value. “NaN” means the method failed to converge to a result within a given amount of time.

For each of the 14×4 experiments, we can obtain a result similar to the example of Table 4. Our interest is to choose the least features to gain the highest accuracy. For example, in Table 4, we get the best performance with the least number of features when we select the top 5 features with k-tuple length 6. We are more interested in the classification performance with a small number of features, so we only considered selection levels below 200. For summarizing the results, we define the “best result” in each experiment as that of the highest accuracy, the lowest feature selection level below the level of 200 and the shortest feature length. The priority order of these criteria is “below 200 features level”, “highest accuracy”, “the lowest feature selection level” and “the shortest feature”.

Table 5 summarizes all results in Sim-1. This set of experiments is designed to study the effect of the type, length and density of seeds in the simulation on the performance of the proposed method, and how many features can give us the best result. We can see that when we set seed density as 0.01, 11 out of the 12 experiments give 100% accuracy while the other one gives a 90% accuracy. When the seed density is 0.005, 7 out of the 12 experiments give 100% accuracy and among the other 5 experiments, 4 of them have accuracies higher than 90%. When the seed density further drops to 0.003, which means that the differences between the two groups are getting very small, still 5 out of the 12 experiments have 100% accuracy, and among the other 7 examples, 6 still have accuracies above 78% and the remaining one has accuracy of 60%, which is the case when the seed is only of 4-letters long.

**Table 5:** Result of simulation 1

**#kind of seeds**	**Density of seed**	**Length = 4**		**Length = 5**		**Length = 6**		**Length = 7**	
		**Best result**	**Accuracy**	**Best result**	**Accuracy**	**Best result**	**Accuracy**	**Best result**	**Accuracy**
0/3	0.003	5-tuple	0.78	5-tuple	0.88	6-tuple	0.82	7-tuple	1.00
		20 features		5f eatures		5 features		5 features	
	0.005	5-tuple	0.76	5-tuple	0.90	6-tuple	1.00	6-tuple	1.00
		50 features		10 features		5 features		5 features	
	0.01	5-tuple	0.90	5-tuple	1.00	5-tuple	1.00	5-tuple	1.00
		20 features		5 features		5 features		5 features	
0/5	0.003	4-tuple	0.86	5-tuple	0.78	6-tuple	1.00	7-tuple	1.00
		200 features		50 features		10 features		5 features	
	0.005	4-tuple	0.94	5-tuple	0.94	6-tuple	1.00	5-tuple	1.00
		100 features		10 features		5 features		5 features	
	0.01	4-tuple	1.00	5-tuple	1.00	5-tuple	1.00	5-tuple	1.00
		5 features		5 features		5 features		5 features	
0/10	0.003	8-tuple	0.60	5-tuple	0.96	6-tuple	1.00	6-tuple	1.00
		50 features		30 features		5 features		5 features	
	0.005	4-tuple	0.96	5-tuple	1.00	6-tuple	1.00	6-tuple	1.00
		100 features		10 features		5 features		5 features	
	0.01	4-tuple	1.00	4-tuple	1.00	4-tuple	1.00	5-tuple	1.00
		20 features		5 features		5 features		5 features	

This table summarizes all the results in simulation 1.

“#kind of seeds” shows how many types of seeds are inserted into the two groups, for example, “0/3” means no seed was inserted in group 1 and 3 kinds of seeds were inserted in group 2. “Length = 4, 5, 6, or 7” means the length of inserted seeds is 4, 5, 6 or 7, respectively.

Table 6 summarizes the results in Sim-2 which was designed to study how the proposed method works when both groups have their own seeds. Only seed density 0.01 was experimented. We can see that 19 out of all 20 experiments can reach the accuracy 100% except for the case of inserting one 4-letters seed in each group which has the accuracy of 96%.

**Table 6:** Result of simulation 2

**#kind of seeds**	**Length = 4**		**Length = 5**		**Length = 6**		**Length = 7**	
	**Best result**	**Accuracy**	**Best result**	**Accuracy**	**Best result**	**Accuracy**	**Best result**	**Accuracy**
1/1	4-tuple	0.96	5-tuple	1.00	5-tuple	1.00	6-tuple	1.00
	5 features		5 features		5 features		5 features	
1/5	4-tuple	1.00	5-tuple	1.00	5-tuple	1.00	6-tuple	1.00
	5 features		5 features		5 features		5 features	
5/5	3-tuple	1.00	5-tuple	1.00	4-tuple	1.00	3-tuple	1.00
	5 features		5 features		5 features		5 features	
5/10	4-tuple	1.00	4-tuple	1.00	4-tuple	1.00	5-tuple	1.00
	5 features		5 features		5 features		5 features	
10/10	3-tuple	1.00	4-tuple	1.00	3-tuple	1.00	4-tuple	1.00
	5 features		5 features		5 features		5 features	

“#kind of seeds” and “best result” have the same meaning with that in Table 4. Seed density is fixed to 0.01.

#### Seed length and feature length

In Sim-1, we changed different seeds and use k-tuples with different lengths as features to study the relation of the result and the underlying information in the sequences. Again, taking the result in Table 4 as an example (with no seed in group 1 and five seeds of length 6 are inserted in group 2), we can see that the best result appears with the k-tuple length of 6 at the 5-feature level. When the number of selected features is 10, 20, 30, or 50, the accuracy is still 100%, but if we select too many features, the accuracy will decrease because most of the selected features are not informative to the classification.

When we use other k-tuples lengths, we may still get 100% accuracy, but not with the least number of features. For example, when we use 5-tuples, the 100% accuracy appears at 20 features level. When we use 7-tuples, the 100% accuracy appears at levels higher than 20 features. From this example and from the summary in Table 5, we can observe that our method attends to give the best result when the length and number of selected features are close to the length and number of inserted seeds.

#### Selected features

**Figure 2:**
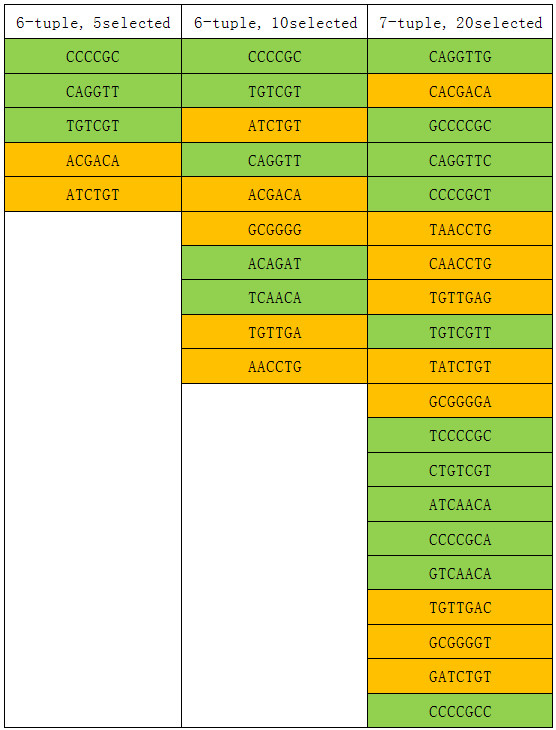
**The selected features from experiment in Table**4**.** This table shows some of the selected features in Table 4's experiment. The inserted seeds in this particular experiment were TGTTGA, ACGACA, AACCTG, GCGGGG and ATCTGT. The first row and the second row show the selected 5 features and 10 features of length 6. The third row shows the selected 20 features of length 7. Feature with yellow shade means it is the seed we inserted, and feature with green shade means it is the seed’s reverse complement sequence.

What features can be revealed by the recursive feature selection and classification method? As an example, Figure 2 shows some of the selected features in the experiment of Table 4. The inserted seeds in this particular experiment were TGTTGA, ACGACA, AACCTG, GCGGGG and ATCTGT. Figure 2 lists the selected 5 features and 10 features with length 6, and the selected 20 features with length 7. We can see that the 5 features and 10 features selected with length 6 are all the inserted seeds or their reverse compliment sequences. (In the Metasim sampling procedure, reads are sampled from both the positive strands and their complimentary strands). Especially, in the selected 10 features of length 6, all the 5 inserted seeds and their reverse compliment sequences are included. At higher selection levels, such as 20 or 30 features with length 6, the selected features all contain these 10 features as well.

The top 20 features with length 7 can also give 100% accuracy. All these 20 selected features contain the seeds or their reverse compliment. When the selection level is too low (too few selected features), even though they can contain the seeds, the feature is one letter longer than the seed so the discrimination information is weakened.

From these simulation experiments, we can see that the feature set selected at the best result or nearly best result can recover the true characteristic differences of the sequence groups.

### Tree family classification

**Figure 3:**
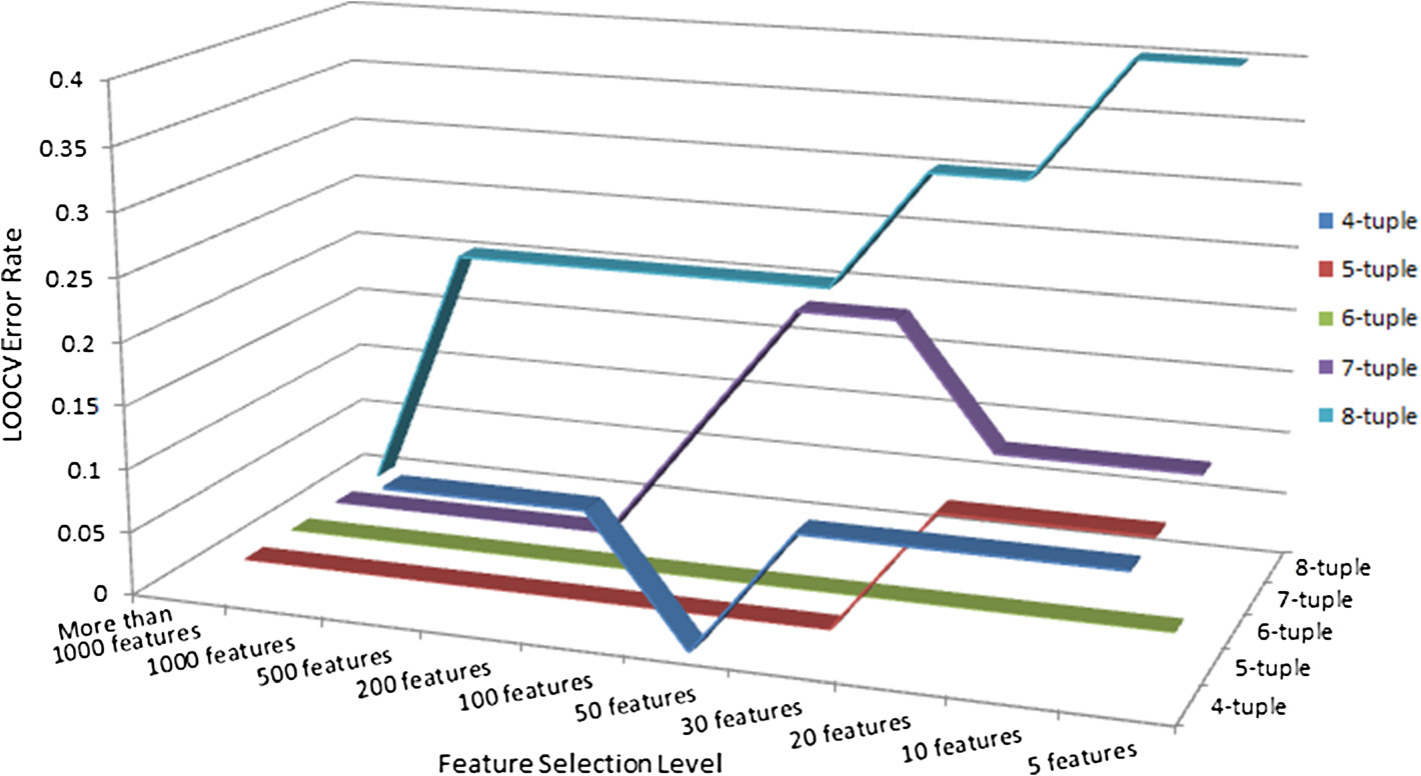
**The LOOCV error rates on real genome data.** This figure shows the LOOCV error rates of different feature lengths and at different feature selection levels on the tree genome data. Each line stands for the LOOCV error rates of one feature length.

We tested our method on the NGS short read data to classify 10 tree genomes into 2 families. Figure 3 shows the LOOCV error rates of different feature lengths and at different feature selection levels. We can see that when we use 6-tuples as features, perfect classification can be obtained at all selection levels, indicating that there are strong distinctions between the two families in their hexamer compositions. In Figure 3, we can also see that the LOOCV accuracy can be very high even when we use all features (especially for the 5-tuple, 6-tuple, 7-tuple features, the accuracy reaches 100% without selection). This can be because that the genome sequence signatures of the two families are very strong. However, even in such a scenario, the recursive feature selection procedure can help us to identify the smallest number of features that can distinguish the two families, and can also help us to identify the most distinctive differences between the compared groups.

We permuted 1000 times on these 10 tree genome data with 6-tuple features, and observed 3 out of the 1000 permutations can result in a 100% accuracy by the same R-SVM experiments. This gives the permutation p-value of 0.003. The reason why 100% accuracy can be achieved on 3 of the 1000 permutated data is that the sample size is too small (only 10 samples) and therefore even some random assignment of class labels may coincide with the true class labels with some probability.

### IBD case classification

**Figure 4:**
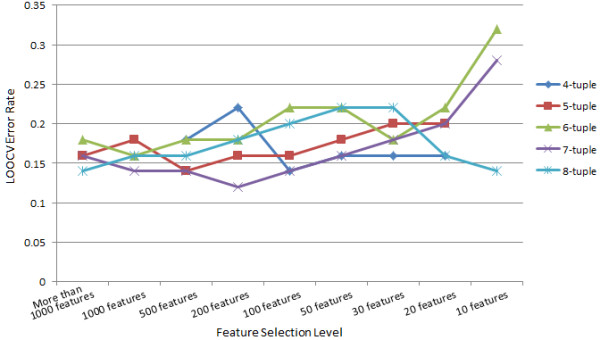
**The LOOCV error rate of real metagenome data.** This figure shows us the LOOCV error rates of different feature lengths and at different feature selection levels on the IBD vs. non-IBD metagenome data. Each line stands for the LOOCV error rates of one feature length.

We applied the proposed method on the real metagenome dataset to classify IBD and non-IBD samples. IBD (Inflammatory Bowel Disease) is an inflammatory disease occurring in the colon or small intestine. It can affect people at any age group and is not easy to treat [44]. People have found indications that it is related with the microbiota in human bowels [44-48]. However, it has not been reported that unsupervised methods on the metagenome data were able to cluster the IBD samples as a group. We had also tried to apply the methods in [33] on this dataset but could not differentiate the IBD samples with other samples using unsupervised methods. This indicates that if there are differences between metagenomes of the IBD patients and control samples, they are not the most observable signal in the data. Therefore we want to see whether metagenome sequence signatures can be used to discriminate IBD patients from normal controls using the supervised approach. We used k-tuple lengths of 4-8 in this experiment. Figure 4 shows the LOOCV error rates of different feature lengths and at different feature selection levels. We can see that the error rate is decreasing when the feature length *k* increases from 4 to 8. When *k* = 7, at the selection level of 200 features, we get the best LOOCV result (accuracy = 88%, sensitivity = 92%, specificity = 84%). This shows that although the IBD samples and control samples cannot be separated by unsupervised clustering with sequence signatures, they can be discriminated by supervised learning methods. This illustrates the power of using alignment-free supervised classification to reveal underlying sequence signatures that distinguish groups of metagenome samples.

**Figure 5:**
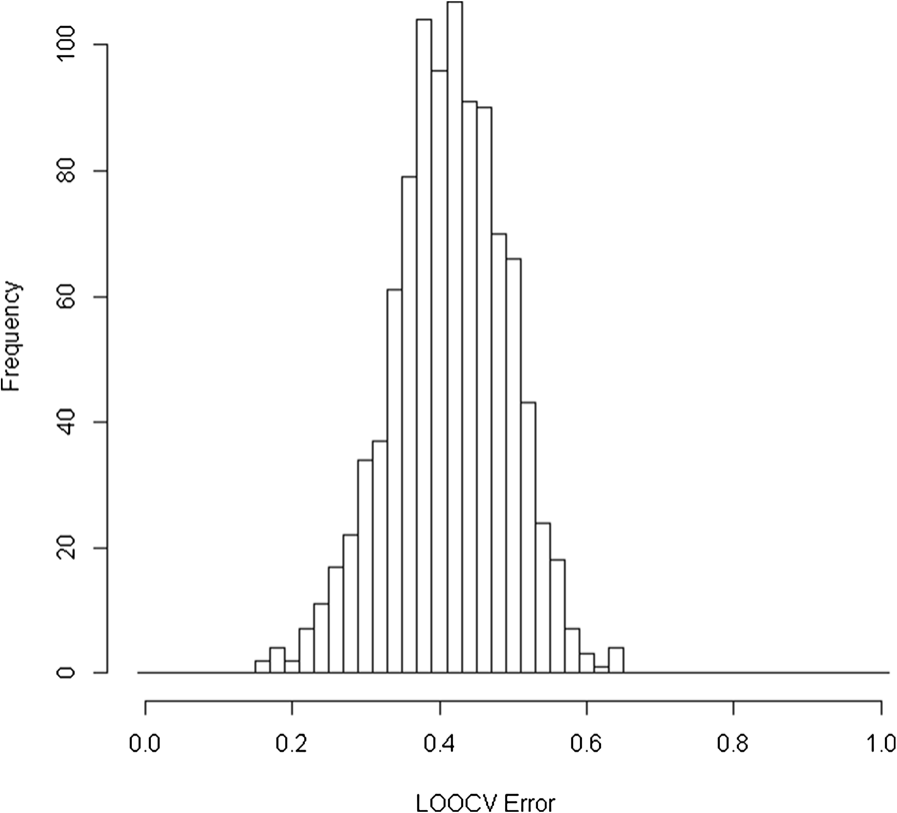
The histogram of the best LOOCV error rates on the permutated IBD data.

We did permutation test to study the significance of this classification result. One thousand permutated datasets were generated and the same R-SVM method was applied on all of them with different k-tuple lengths as on the real data to choose a best LOOCV result for each permutation dataset. None of the 1000 permutations can give us the accuracy equal to or higher than 88%, which gives the p-value less than 0.001. Figure 5 shows the distribution of all the LOOCV error rates of 1000 permutations in the IBD case experiments. We can see that all the LOOCV error rates on the permuted data are distributed between 16% and 64% and most of them are between 30-50%. This shows that although the classification of IBD vs. non-IBD samples with metagenome sequence features is not as ideal as the simplified examples in the above experiments, the achieved accuracy are statistically highly significant. This is a strong evidence that IBD is associated with metagenome features.

After the LOOCV experiment, we re-run the R-SVM method on all 50 training samples with k-tuple length of 7 to obtain a classifier to be applied for predicting other samples. We applied the classifier on the remaining 74 samples (all non-IBD) as the independent test data and found 78% of them are correctly predicted.

We did 4 extra experiments by randomly choosing a different group of 25 control samples in the training data each time. Table 7 summarizes the LOOCV accuracies and test accuracies in all the 5 experiments.

**Table 7:** The LOOCV and test accuracies of the 5 experiments on the IBD data

	**Experiment 1**	**Experiment 2**	**Experiment 3**	**Experiment 4**	**Experiment 5**
LOOCV Acc	88%	86%	88%	90%	86%
Test Acc	78%	84%	78.4%	73%	86.5%

## Conclusions

In this paper, we developed an alignment-free supervised classification approach to classify metagenome samples into predefined classes with sequence signatures from NGS data. We conducted a series of simulations to study the performance of the method and effects of parameters. Simulation results show that the method is powerful in classifying the classes, and can successfully reveal underlying features that distinguish the two classes. Then we applied the method to a real genome dataset and a real metagenome dataset to test its ability to handle real NGS sequences. It can give 100% LOOCV accuracy on the genome dataset and ~88% LOOCV accuracy on the metagenome dataset. These results are proved to be significant by permutation test.

The rapidly developing next-generation sequencing technologies provided great opportunity for studying the microbial communities of different environments or human niches. The lacking of sufficient reference genomes and also the computational burden of assembling microbial genomes from metagenome data hinders the wider application of such technology. Experiments in this study showed that these problems can be solved by adopting the alignment-free strategy together with machine learning methods. Sequence signatures can be analyzed directly on the short reads, and the biological functions can be studied by downstream analyses of sequence signatures that are selected as discriminative for the classification.

The method R-SVM adopted in this work is a representative for supervised classification and feature selection methods. There are many other optional methods that can be applied, such as the Naive Bayes method, k-nearest neighbor method, decision tree and random forest, etc.. These method need to be combined with some feature selection methods for the application. R-SVM represents a category of methods that integrate the feature selection and classification steps in one “wrapper” method. Brown et al discussed that “wrapper methods have higher learning capacity so are more likely to overfit” [49]. Extra caution must be made when designing such experiments. Including the feature selection step inside the cross-validation procedures is crucial for avoiding overfitting and biased assessment of the performance [39,50]. And the permutation strategy can be used to check the significance of the observed accuracy by estimating the p-value of getting the result by chance due to the high capacity of the method.

A current limitation of the alignment-free methods is that after a set of sequence signatures are found to be discriminative for the classification, the biological indication of these k-tuple features is still not clear. This is a limitation for both supervised and unsupervised methods. The biological meaning of selected k-tuple sequence signatures is still an open question for future study. Any breakthrough on this direction may open a new gate for understanding functions of microbiomes and their interaction with the host systems they parasitize or accrete with.

## Abbreviations

PCA: Principal component analysis; NGS: Next-generation sequencing; R-SVM: Recursive support vector machines; LOOCV: Leave-one-out cross validation; IBD: Inflammatory bowel disease; RDP: Ribosomal database project; OTU: Operational taxonomic unit; NCBI: National center for biotechnology information

## Competing interests

The authors declare that they have no competing interests.

## Authors’ contributions

XZ initiated and designed the study. HC did all the experiments. HC and XZ analyzed the results and wrote the manuscript. Both authors read and approved the final manuscript.
